# Spin currents and torques in ferromagnetic systems with strong interfacial spin-orbit coupling

**DOI:** 10.1038/s41598-025-22567-1

**Published:** 2025-11-05

**Authors:** Nils Petter Jørstad, Bernhard Pruckner, Wolfgang Goes, Siegfried Selberherr, Viktor Sverdlov

**Affiliations:** 1 Christian Doppler Laboratory for Nonvolatile Magnetoresistive Memory and Logic, Vienna, Austria; 2 Institute for Microelectronics, TU Wien, Gußhausstraße 27-29, 1040 Vienna, Austria; 3 Silvaco Europe Ltd., Compass Point, St Ives, Cambridge, PE27 5JL UK

**Keywords:** Spin-orbit coupling, Spin-orbit torque, Rashba-Edelstein effect, Spin currents, Ferromagnetic trilayers, Magnetic properties and materials, Spintronics

## Abstract

A three-dimensional description of spin-dependent current transport across nonmagnetic/ferromagnetic interfaces with strong interfacial spin-orbit coupling is presented. The resulting current-induced torques acting on the magnetization of the ferromagnetic layer are addressed. By considering both magnetic exchange and Rashba spin-orbit interactions at the interface, the angular dependence of the spin-orbit torques in Pt/Co and Ta/CoFeB systems is reproduced. In line with two-dimensional Rashba models, the Rashba-Edelstein effect drives the strong field-like torque, with the unconventional angular dependence being most pronounced, when magnetic exchange and spin-orbit interaction strengths are comparable. Furthermore, the spin currents generated through spin-orbit precession and filtering are shown to produce all three spin-polarization components, depending on the magnetization direction. Utilizing these mechanisms in current-in-plane trilayers could potentially enable field-free perpendicular and type-x switching, which is crucial for advancing the miniaturization of spintronic devices.

## Introduction

In systems lacking inversion symmetry, such as zinc-blende crystals, two-dimensional materials, and interfaces, the spin degeneracy in electronic energy bands is lifted by the spin-orbit coupling (SOC)^1^. Under an applied electric field, the occupation of the bands is biased, resulting in a nonequilibrium spin accumulation^2^. A similar mechanism in bulk materials with SOC allows for an unpolarized current to be converted to a pure spin current^3^. These effects are respectively known as the Rashba-Edelstein effect (REE) and the spin Hall effect (SHE), and are considered to be the primary phenomena responsible for the torque acting on the magnetization in current-in-plane (CIP) nonmagnetic (NM)/ferromagnetic (FM) bilayers, which are collectively known as spin-orbit torques (SOTs) due to their shared SOC origin^4–6^.

**Fig. 1:**
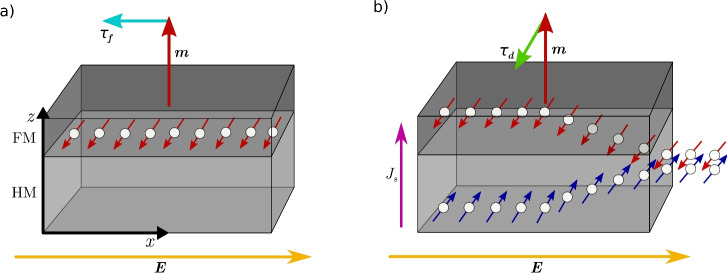
A sketch depicting the spin accumulation generation in CIP NM/FM bilayers through the REE (**a**) and the SHE (**b**). In both cases, an electric field $\varvec{E}$ along $\varvec{x}$ drives a spin-polarized current. The generated nonequilibrium spin accumulation is polarized along $\varvec{E}\times \varvec{\hat{z}}$. The field-like and damping-like torques are attributed to the REE and SHE, respectively.

In the bulk of the NM layer, the SHE generates a spin current impinging on the FM layer. Concurrently, at the interface, the REE generates a net spin accumulation. The spin accumulation in the FM layer, resulting from both effects, has an in-plane polarization along $(\varvec{E}\times \varvec{\hat{z}})$, where $\varvec{E}$ is the electric field and the $\varvec{\hat{z}}$ is perpendicular to the interface. A sketch of the system is depicted in Fig. 1. In the FM layer, angular momentum is transferred from the spin accumulation to the magnetization through the spin-transfer mechanism, which the magnetization experiences as a current-induced torque, enabling efficient manipulation of the magnetic state. These torques can be decomposed into a field-like (FL) component describing the precession of the magnetization around the direction of the spin accumulation: $\varvec{\tau _f} = \tau _f\varvec{m}\times (\varvec{E}\times \varvec{\hat{z}})$, and a damping-like (DL) component describing the damping towards it: $\varvec{\tau _d} = \tau _d\varvec{m}\times \left[ \varvec{m}\times (\varvec{E}\times \varvec{\hat{z}})\right]$, where $\varvec{m}$ is the magnetization direction, and $\tau _f$ and $\tau _d$ are the magnitudes of the torque components. The FL torque is predominantly attributed to the REE, while the DL torque is primarily attributed to the SHE. Yet, both effects can contribute to both components of the torque^4^.

The discovery of SOTs has enabled efficient and scalable control of magnetic states using applied currents, leading to the development of several SOT-based devices^5–10^. For instance, spin Hall nano-oscillators (SHNOs) use SOTs to drive an oscillating magnetization in a FM layer, producing an oscillating electric voltage. SHNOs can convert direct current inputs into alternating current signals over a wide frequency range, making them promising candidates for applications in wireless communication and neuromorphic computing^11,12^. Another promising application is using SOTs for writing in nonvolatile magnetoresistive random access memory (MRAM). In MRAM, logical states are stored based on the relative magnetization orientation of two FM layers in a magnetic tunneling junction (MTJ). Typically, spin-transfer torques (STT) are used to write the state in MRAM, which requires a considerable current perpendicular to the plane, degrading the MTJ. In contrast, SOT-MRAM features an in-plane write path below the MTJ, significantly increasing endurance, albeit at the cost of having three terminals instead of two. MRAM offers several advantages over conventional memory technologies, primarily eliminating the need for a standby current, which promises considerable improvements in energy efficiency. However, advancing SOT-MRAM to the point where it can replace standard memory technologies presents numerous challenges^9,13,14^. This necessitates the development of computationally efficient models that can accurately capture the key physical phenomena driving SOTs.

Most descriptions of SOT in CIP bilayers only account for the conventional DL and FL torques. However, symmetry allows for a more complicated dependence on the magnetization direction, which has been confirmed by experiments and *ab initio* calculations^15–17^. Previous models based on the REE effect in two-dimensional ferromagnetic systems have accounted for the strong dependence on the magnetization direction^18,19^, showing that the angular dependence appears, when the SOC and exchange coupling are comparable in strength. However, NM/FM bilayers require a three-dimensional description to capture the spin and charge currents generated in the bulk of each layer and their interaction with the interface. One proposed approach treats the SOC as a perturbation localized at the interface with perturbation theory^20^, which is limited to a weak SOC compared to the exchange coupling and thus cannot account for the reported angular dependence. Another approach treats the interface with a spin- and momentum-dependent delta function potential barrier^21^, which allows for the investigation of the strong SOC regime.

In this work, we present boundary conditions obtained by considering the latter approach. In the spirit of the magnetoelectronic circuit theory (MCT), we describe the interface currents in terms of reflected and transmitted distribution functions^22,23^, which results in momentum-averaged interface conductance and conductivity tensors. To account for the interaction of the out-of-plane currents from the bulk with the interface, we employ drift-diffusion equations and use the expressions for the currents as boundary conditions. The inclusion of a momentum-dependent field at the interface gives rise to spin-generating mechanisms unique to the three-dimensional approach and a generalization of the REE accounting for both the in-plane charge and spin currents^24^. We describe the torques acting on the magnetization at the interface and identify the new contributions arising from the interfacial SOC. We compare our results with the experimental literature and investigate the dependence of the torque on the interfacial exchange and Rashba spin-orbit interactions. Furthermore, we investigate the role of the contributions arising from the SOC and the modification of the torques from the MCT. Finally, we investigate using the interface-generated currents in FM/NM/FM trilayers for deterministic field-free switching.

## Interface model

**Fig. 2:**
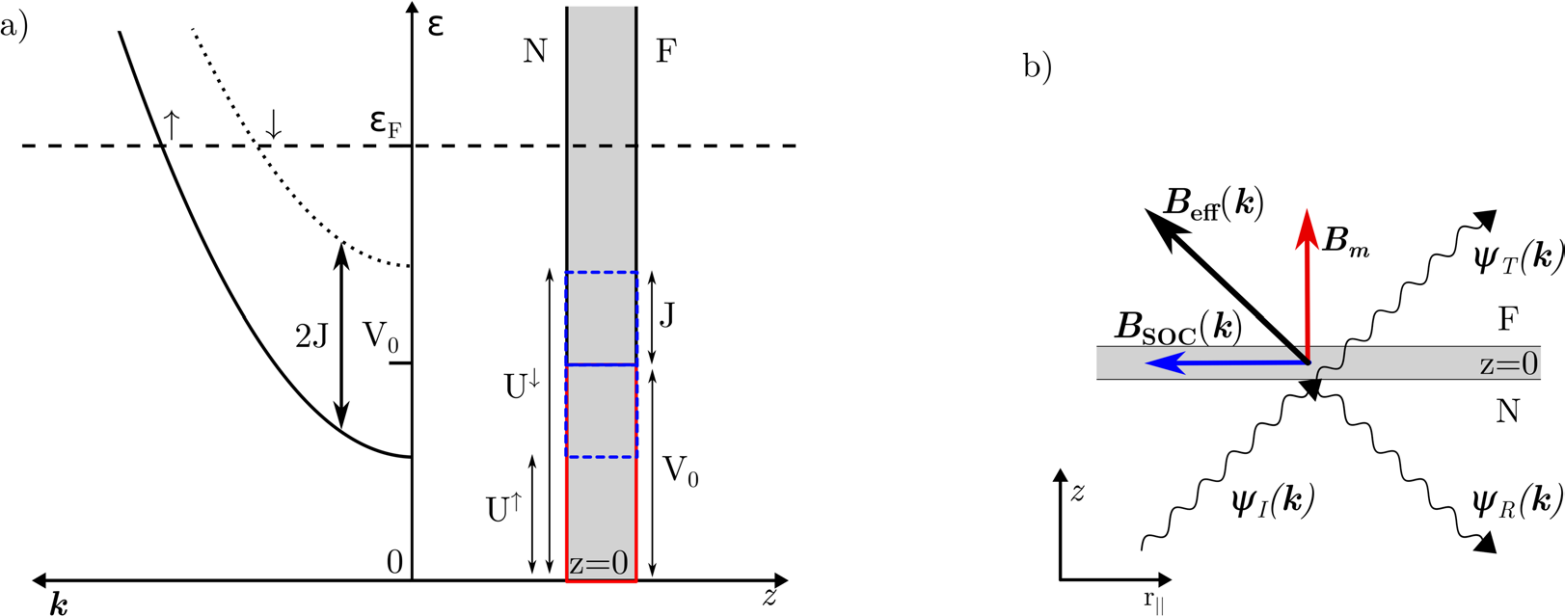
(**a**) A schematic of the interface model. The carriers experience different potential barriers $U^{\uparrow /\downarrow } = V_0 \mp J_\textrm{ex}$ at the interface depending on their spin. In the zero-thickness interface region, the carriers are split into majority and minority bands, depending on their spin being antiparallel or parallel to the field at the interface, respectively. (**b**) A schematic of the scattering process. A wave function incident on an interface splits into a reflected and transmitted part. The effective interface field comprises an interfacial magnetization exchange field and a momentum-dependent spin-orbit field.

We consider an interface at $z=0$ separating a FM layer ($z > 0$) from a NM layer ($z < 0$), with an effective magnetic field $\varvec{B}$ located at the interface. For simplicity, we assume that the exchange splitting in the bulk of the FM layer is weak relative to that at the interface. Therefore, we characterize the bulk on both interface sides as a spin-independent free electron gas. The interface is described with a delta function potential barrier containing a spin-independent and spin-dependent part. The scattering system is depicted in Fig. 2. The Hamiltonian describing this system is given by^21^

1 $$\begin{aligned} \hat{H}=-\frac{\hbar ^2}{2 m_e} \nabla ^2+ \delta (z)\left( V_0\hat{1} + J_\textrm{ex}\varvec{\hat{\sigma }}\cdot \varvec{b} \right) , \end{aligned}$$

where $\hbar$, $m_e$, and $\nabla$ are the reduced Planck’s constant, electron mass, and Nabla operator, respectively. Hat ($^\mathrm{\wedge }$) denotes a $2\times 2$ matrix in spin-space, $\delta (z)$ is the Dirac delta function, and $\varvec{\hat{\sigma }}$ is the vector of the Pauli matrices. The spin-independent potential energy is $V_0 = \hbar ^2k_F u_0/m_e$, while $J_\textrm{ex} = \hbar ^2k_F u_{ex}/m_e$ is the effective interfacial exchange energy, where $u_0$ and $u_\textrm{ex}$ are the dimensionless magnitudes of each potential and $k_F$ is the fermi wave number. The vector $\varvec{b}= \varvec{B}/B$ is the direction of the effective magnetic field at the interface. To be consistent with other models the majority ($\uparrow$) and minority ($\downarrow$) electrons have a spin antiparallel ($\sigma = -1)$ and parallel ($\sigma = +1)$ to the magnetic field, respectively. There is no explicit difference between either side of the interface in the Hamiltonian; the difference is instead captured by the nonequilibrium distribution functions at either side of the interface.

By considering plane-wave solutions of the time-independent Schrödinger equation and in-plane-momentum-conserving scattering, the reflection and transmission coefficients for majority/minority carriers read^24^

2 $$\begin{aligned} r_{\varvec{k}}^{\uparrow / \downarrow } =\frac{u^{\uparrow /\downarrow }}{ i (k_z/k_F)-u^{\uparrow /\downarrow }}, \quad \text {and}\quad t_{\varvec{k}}^{\uparrow / \downarrow } =\frac{ i (k_z/k_F)}{ i (k_z/k_F)-u^{\uparrow /\downarrow }}, \end{aligned}$$

respectively, where $u^{\uparrow /\downarrow } = u_0 \mp u_{ex}$ is the dimensionless magnitude of the potential barrier for majority/minority carriers. The scattering matrices in Pauli spin space are then given by

3 $$\begin{aligned} \hat{r}_{\varvec{k}} = \sum _s \hat{p}^s r_{\varvec{k}}^s,\quad \text {and}\quad \hat{t}_{\varvec{k}} = \sum _s \hat{p}^s t_{\varvec{k}}^s \end{aligned}$$

for $s\in \{\uparrow ,\downarrow \}$, where $\hat{p}^{\uparrow /\downarrow } = (\hat{1} \mp \varvec{\hat{\sigma }}\cdot \varvec{b})/2$ is the spin projection matrix for majority/minority carriers.

Without affecting the results up to this point, we assume that the effective magnetic field at the interface is momentum-dependent, i.e., $\varvec{b}\rightarrow \varvec{b}_{\varvec{k}}$, and consequently $u^{\uparrow /\downarrow }\rightarrow u^{\uparrow /\downarrow }_{\varvec{k}}$, $\hat{p}^{\uparrow /\downarrow }\rightarrow \hat{p}^{\uparrow /\downarrow }_{\varvec{k}}$. Furthermore, we assume that it can be split into two contributions: a momentum-independent interfacial exchange interaction between the electron’s spin and the magnetization at the interface with energy $J_m =\hbar ^2k_F u_{m}/m_e$, and a spin-orbit field along ${\varvec{b}_{\textrm{SO},\varvec{k}}}$ with the exchange energy $J_\textrm{SO}=\hbar ^2k_F u_\textrm{SO}/m_e$, where $u_m$ and $u_\textrm{SO}$ are the respective dimensionless interaction magnitudes. Thus, the direction of the effective field is given by $\varvec{b_k} = (J_m\varvec{m} + J_\textrm{SO}{\varvec{b}_{\textrm{SO},\varvec{k}}})/J_\textrm{ex}$, with $J_\textrm{ex} = \Vert J_m\varvec{m} + J_\textrm{SO}{\varvec{b}_{\textrm{SO},\varvec{k}}}\Vert$. In principle, any spin-orbit field can be incorporated into the effective field at the interface, such as the Dresselhaus or Rashba spin-orbit fields.

## Out-of-plane charge and spin currents

The expressions derived in this and the following section are analogous to the generalized circuit theory formulated by Amin & Stiles using a Boltzmann equation approach to describe the NM/FM interface^25,26^, therefore, we adopt their terminology and notation to some extent to draw attention to the similarities. The main difference is that our approach allows to separate the currents into the individual contributions arising from the out-of-plane and in-plane charge and spin currents, facilitating the identification of mechanisms and modifications introduced by the interfacial SOC, which is crucial for our analysis.

The out-of-plane current densities on the NM ($0^-$) and FM ($0^+$) sides of the interface can be described in terms of local distribution functions in the NM and FM layer $\hat{f}^N_{\varvec{k}}$ and $\hat{f}^F_{\varvec{k}}$, respectively. Similarly to the Landauer-Büttiker formalism, the currents are then determined by the incident, reflected, and transmitted distribution functions, where the latter two are described by the reflection and transmission matrices from the previous section, respectively. The energy resolved out-of-plane current densities at either side of the interface can be expressed as^22,23^

4 $$\begin{aligned} \hat{j}_z(0^\pm ,\varepsilon _{\varvec{k}})=\frac{\pm e}{2\pi \hbar A} \sum _{\varvec{k_\Vert }} \left[ \hat{f}_{\varvec{k}}^{\mathrm {F/N}} -\hat{r}_{\varvec{k}} \hat{f}_{\varvec{k}}^{\mathrm {F/N}} \hat{r}_{\varvec{k}}^{\dagger }-\hat{t}_{\varvec{k}} \hat{f}_{\varvec{k}}^{\mathrm {N/F}} \hat{t}_{\varvec{k}}^{\dagger } \right] , \end{aligned}$$

where *A* is the surface area of the interface and $\varepsilon _{\varvec{k}}$ is the energy of a state with momentum $\varvec{k}$.

In equilibrium, the reduced density matrix is diagonal in spin space, i.e., $\hat{f}_{\varvec{k},\textrm{eq}}^{N/F}= \hat{1}f_{\varvec{k},\textrm{eq}}^{N/F}$, where $f_{\varvec{k},\textrm{eq}}^{N/F}$ is the Fermi-Dirac distribution. In the linear response regime, the out-of-equilibrium distributions are given by

5 $$\begin{aligned} \hat{f}^{N/F} = \hat{f}_{\varvec{k},eq}^{N/F} + \frac{\partial \hat{f}_{\varvec{k},eq}^{N/F}}{\partial \epsilon _{\varvec{k}}}\hat{g}^{N/F}_{\varvec{k}}, \end{aligned}$$

where $\hat{g}^{N/F}_{\varvec{k}}$ is the nonequilibrium distribution function. Because of the unitarity relation of the scattering matrices:$\hat{1} - \hat{r}_{\varvec{k}}(\hat{r}_{\varvec{k}})^\dagger = \hat{t}_{\varvec{k}}(\hat{t}_{\varvec{k}})^\dagger$, only the nonequilibrium part of the distribution function contributes to the current.

The total current densities are obtained by integrating over the energies: $\hat{J}_z(0^\pm ) = \int d\epsilon \hat{j}_z(0^\pm ,\varepsilon )$. In the zero temperature limit, we can approximate $\partial \hat{f}_{\varvec{k},eq}^{N/F}/\partial \epsilon _{\varvec{k}} \approx \hat{1}\delta (\epsilon _{\varvec{k}}-\epsilon _F)$, where $\epsilon _F = \hbar ^2k_F^2/2m_e$ is the Fermi energy. After evaluating the integral over the energy and expressing the scattering matrices in terms of the spin projection matrices, we obtain

6 $$\begin{aligned} \hat{J}_z(0^\pm )= & \frac{1}{eA}\sum _{\varvec{k_\Vert }\in \textrm{FS}} \left[ {\mathcal {G}}^{\uparrow \uparrow }_{\varvec{k}}\hat{p}_{\varvec{k}}^\uparrow \Delta \hat{g}_{\varvec{k}}\hat{p}_{\varvec{k}}^\uparrow + {\mathcal {G}}^{\downarrow \downarrow }_{\varvec{k}}\hat{p}_{\varvec{k}}^\downarrow \Delta \hat{g}_{\varvec{k}}\hat{p}_{\varvec{k}}^\downarrow \pm {\mathcal {G}}^{\uparrow \downarrow }_{\varvec{k}}\hat{p}_{\varvec{k}}^\uparrow \hat{g}_{\varvec{k}}^{\mathrm {F/N}}\hat{p}_{\varvec{k}}^\downarrow \pm ({\mathcal {G}}^{\uparrow \downarrow }_{\varvec{k}})^*\hat{p}_{\varvec{k}}^\downarrow \hat{g}_{\varvec{k}}^{\mathrm {F/N}}\hat{p}_{\varvec{k}}^\uparrow \mp {\mathcal {T}}^{\uparrow \downarrow }_{\varvec{k}}\hat{p}_{\varvec{k}}^\uparrow \hat{g}_{\varvec{k}}^{\mathrm {N/F}}\hat{p}_{\varvec{k}}^\downarrow \right. \nonumber \\ & \left. \mp ({\mathcal {T}}^{\uparrow \downarrow }_{\varvec{k}})^*\hat{p}_{\varvec{k}}^\downarrow \hat{g}_{\varvec{k}}^{\mathrm {N/F}}\hat{p}_{\varvec{k}}^\uparrow \right] , \end{aligned}$$

where $\Delta \hat{g}_{\varvec{k}} = \hat{g}^F_{\varvec{k}}-\hat{g}^N_{\varvec{k}}$. Here, we have introduced the momentum-resolved spin-dependent conductances ${\mathcal {G}}^{ss^\prime }_{\varvec{k}} = G_0[1 - r^{s}_{\varvec{k}}(r_{\varvec{k}}^{s^\prime })^*]$ and ${\mathcal {T}}^{ss^\prime }_{\varvec{k}} = G_0[t^{s}_{\varvec{k}}(t_{\varvec{k}}^{s^\prime })^*]$, for $s, s^\prime \in \{\uparrow ,\downarrow \}$, where $G_0 = e^2/(2\pi \hbar )$. The majority and minority conductances ${\mathcal {G}}^{\uparrow \uparrow }_{\varvec{k}}$ and ${\mathcal {G}}^{\downarrow \downarrow }_{\varvec{k}}$, respectively, describe the collinear transport, while the spin-mixing conductances ${\mathcal {G}}^{\uparrow \downarrow }_{\varvec{k}}$ and ${\mathcal {T}}^{\uparrow \downarrow }_{\varvec{k}}$ describe the noncollinear transport of spins with momentum $\varvec{k}$. The summation over the in-plane wave vectors is limited to the Fermi sphere, such that $k_\Vert = \sqrt{k_F^2 -k_z^2}$.

The nonequilibrium distribution function can be separated into an isotropic nonequilibrium chemical potential and an anisotropic ”drift” term describing the deformation of the Fermi-surface by the applied field^23,27^:

7 $$\begin{aligned} \hat{g}^{N/F}_{\varvec{k}} = e\hat{\mu }^{N/F}+e\hat{\gamma }^{N/F}_{\varvec{k}} \end{aligned}$$

The anisotropic term is often ignored, as it vanishes when averaging the distribution over the Fermi surface; however, this is not the case when the effective magnetic field is momentum-dependent. The shift of the Fermi-surface by an applied electric field along *x* in the FM and NM layer can be expressed as

8 $$\begin{aligned} \hat{\gamma }^{F}_{\varvec{k}} = v_F \frac{k_x}{k_F}\tau ^FE_x\left( \hat{1}-\varvec{\sigma }\cdot \varvec{m}P\right) , \quad \text {and}\quad \hat{\gamma }^{N}_{\varvec{k}} = v_F \frac{k_x}{k_F}\tau ^NE_x\hat{1}, \end{aligned}$$

respectively, where $\tau ^{F/N}$ is the momentum relaxation time of each layer, $v_F = \hbar k_F/m_e$ is the Fermi velocity, and *P* is the polarization of the current in the FM layer. The chemical potential can also be expanded in Pauli matrices such that $e\hat{\mu } = e\hat{1}\mu _c + e\varvec{\hat{\sigma }}\cdot \varvec{\mu _s}$, where $\mu _c$ and $\varvec{\mu _s}$ are the net charge and spin accumulations in units of voltage, respectively.

The charge and spin current densities in real space are given by $J_{zc}(0^\pm )= \operatorname {Tr}_{\sigma }[\hat{J}_z(0^\pm )]$ and $\varvec{J_{zs}}(0^\pm )= \operatorname {Tr}[\varvec{\hat{\sigma }} \hat{J}_z(0^\pm )]$, respectively, where $\operatorname {Tr}[\ ]$ is the trace over the spin components. Inserting Eq. (7) into Eq. (6), and performing the traces, the momentum dependence can be factorized, yielding momentum-averaged conductance and conductivity tensors. Following these steps, the charge and spin currents at the interface read

9a $$\begin{aligned} J_{zc}(0^\pm )&= G_{+}\Delta \mu _{c} - \varvec{G_{-}}\cdot \Delta \varvec{\mu _{s}} + \Delta \sigma _+ E_x + P(\varvec{\sigma _{-}^F}\cdot \varvec{m})E_x,\end{aligned}$$

9b $$\begin{aligned} {\varvec{J}_{zs}}(0^+)&= \tilde{G}_{+}\Delta \varvec{\mu _{s}} -\varvec{G_{-}}\Delta \mu _{c} - \tilde{G}_{\uparrow \downarrow }\varvec{\mu _{s}^{\textrm{F}}} + \tilde{\Gamma }_{\uparrow \downarrow }\varvec{\mu _{s}^{\textrm{N}}} -\Delta \varvec{\sigma _{-}}E_x -P(\tilde{\sigma }^F_{+}-\tilde{\sigma }^F_{\uparrow \downarrow })\varvec{m}E_x, \text { and } \end{aligned}$$

9c $$\begin{aligned} {\varvec{J}_{zs}}(0^-)&= \tilde{G}_{+}\Delta \varvec{\mu _{s}} -\varvec{G_{-}}\Delta \mu _{c} + \tilde{G}_{\uparrow \downarrow }\varvec{\mu _{s}^{\textrm{N}}} - \tilde{\Gamma }_{\uparrow \downarrow }\varvec{\mu _{s}^{\textrm{F}}} -\Delta \varvec{\sigma _{-}}E_x -P(\tilde{\sigma }^F_{+}-\tilde{\gamma }^F_{\uparrow \downarrow })\varvec{m}E_x, \end{aligned}$$

where $\Delta \mu _{c} = (\mu _{c}^{\textrm{F}}-\mu _{c}^{\textrm{N}})$ and $\Delta \varvec{\mu _{s}} = (\varvec{\mu _{s}^{\textrm{F}}}-\varvec{\mu _{s}^{\textrm{N}}})$ are the charge and spin accumulation drops across the interface, respectively. Similarly, $\Delta \sigma _+ = (\sigma _+^{\textrm{F}}-\sigma _+^{\textrm{N}})$ and $\Delta \varvec{\sigma _{-}} = (\varvec{\sigma _{-}^{\textrm{F}}}-\varvec{\sigma _{-}^{\textrm{N}}})$ are the differences in the interface conductivities. A tilde above a quantity denotes that it is a $3\times 3$ matrix. The details of the momentum-averaged conductances and conductivities are presented in the appendix. The spin current is given here in units of A/m$^2$ and can be converted into angular momentum current density units by multiplication with $\hbar /2e$.

**Fig. 3:**
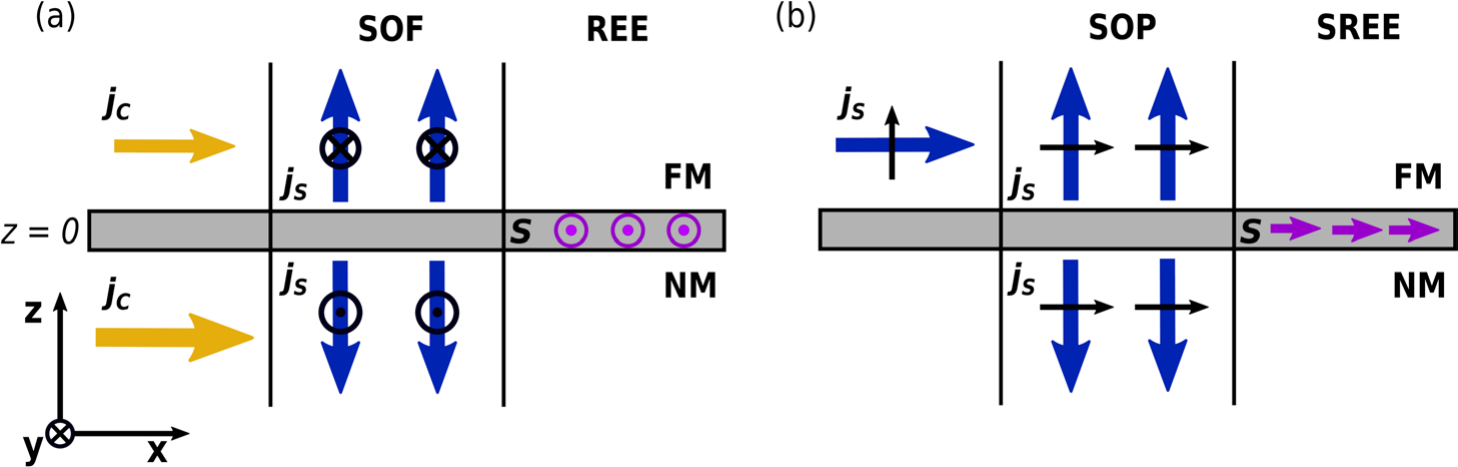
A schematic illustration of the SOF, SOP, REE, and SREE mechanisms in CIP NM/FM bilayers for the case of Rashba-type SOC at the interface and a magnetization direction along the *z* axis. (**a**) In-plane charge currents (yellow arrows) on either side of the interface couple to the interfacial SOC, producing out-of-plane spin currents (blue arrows) via the SOF and an interfacial spin density (purple arrows) via the REE. The black arrows show the polarization direction of the spin currents. (**b**) In-plane spin currents in the FM layer give rise to out-of-plane spin currents through SOP and generate an interfacial spin density via the SREE.

The first two terms in Eq. (9a), and the first four terms in Eqs. (9b) and (9c), describe the out-of-plane charge and spin currents due to the charge and spin accumulation drops across the interface. These terms capture the interaction between the out-of-plane bulk currents in the NM and FM layers with the interface. In the limit of vanishing SOC, they are analogous to the interface currents described by the MCT, except for the fourth term in Eqs. (9b) and (9c), which is the transverse spin current transmitted across the interface. The fifth and sixth terms in Eqs. (9b) and (9c) describe spin currents generated by the in-plane charge and spin currents, which originate from the spin-orbit filtering (SOF) and spin-orbit precession (SOP) mechanisms^24^, respectively. SOF results from the SOC at the interface acting as a spin- and momentum-dependent filter, such that an unpolarized stream of electrons becomes spin-polarized through the scattering process at the interface. SOP occurs when an incident spin-polarized stream of electrons changes its polarization direction by precessing around the SOC field throughout the scattering process. The third and fourth terms in Eq. (9a) describe the reciprocal process, where the in-plane charge and spin currents generate out-of-plane charge currents. The transmitted polarized electron streams from the SOF and SOP mechanisms both generate a non-equilibrium spin-density at the interface, which is analogous to the REE in this model when the SOC is of the Rashba form. Figure 3 illustrates the spin currents and spin densities generated by the in-plane currents interacting with the interfacial SOC in the case of a Rashba SOC and $u_m = 0$. The polarization of the SOF-generated spin density is along ${\textbf{E}}\times {\textbf{z}}$, consistent with conventional 2D REE models; we therefore identify this contribution as the REE. By contrast, the SOP-induced spin density is polarized along $({\textbf{E}}\times {\textbf{z}})\times {\textbf{m}}$. Because this contribution has a distinct polarization direction and originates from the in-plane spin current in the FM layer, we refer to it as the spin Rashba–Edelstein effect (SREE), distinguishing it from the conventional REE driven by in-plane charge currents.

We employ drift-diffusion equations for spin and charge to describe the bulk currents and accumulations. For simplicity, we assume that the width and length of the system are significantly larger than the thicknesses of the layers and that the system is homogeneous in the *xy* plane. Thus, we will only consider the out-of-plane currents. Moreover, we assume that a constant in-plane electric field $\varvec{E} \Vert \varvec{\hat{x}}$ is applied across all the layers and consider the polarization of the currents in the FM layers and the spin Hall effect in the NM layers. The out-of-plane spin and charge currents in the NM and FM can then be expressed as^21,26^

10a $$\begin{aligned} {\varvec{J}_{zs}}(z) = - \sigma \partial _z\varvec{\mu _s}(z) + P\sigma \varvec{m} \partial _z\mu _c(z) + \alpha _\textrm{SH}\sigma \varvec{E}\times \varvec{\hat{z}}\end{aligned}$$

10b $$\begin{aligned} J_{zc}(z) = -\sigma \partial _z\mu _c(z) + P\sigma \varvec{m}\cdot \partial _z\varvec{\mu _s}(z), \end{aligned}$$

where $\sigma$ is the electrical conductivity, and $\alpha _\textrm{SH}$ is the spin Hall angle. In the NM and FM layer, $P = 0$ and $\alpha _\textrm{SH} = 0$, respectively.

The steady-state continuity equations for the spin and charge currents read^21,26^

11a $$\begin{aligned} \partial _z {\varvec{J}_{zs}}(z)&= -\sigma \left[ \frac{\varvec{\mu _s}(z) }{\lambda _{sf}^2} +\frac{\varvec{\mu _s}(z) \times \varvec{m}}{\lambda _{J}^2}+\frac{\varvec{m}\times (\varvec{\mu _s}(z) \times \varvec{m})}{\lambda _{\phi }^2}\right] , \end{aligned}$$

11b $$\begin{aligned} \partial _z J_{zc}(z)&= 0, \end{aligned}$$

where $\lambda _{sf}$, $\lambda _{J}$, and $\lambda _{\phi }$ are the spin-flip, exchange, and dephasing lengths, respectively. The spin-flip length is the characteristic distance over which the electron’s spin loses its original orientation due to bulk spin-flip scattering. The spin-exchange and dephasing lengths characterize the distance over which the transverse spin accumulation is transferred to the local magnetic moments due to the bulk exchange interaction and dephasing process, respectively.

The spin current generated by the SHE in the bulk is influenced by the interfacial SOC in several ways. First, the SOP and SOF spin currents produced on the NM side of the interface can interfere constructively or destructively with the SHE spin current, effectively modifying the spin Hall angle. Second, the interaction of the SHE spin current with the NM/FM interface, typically described by the complex mixing conductance that captures the rotation of the spin around the magnetization, is altered by the presence of SOC. The interfacial SOC causes an additional rotation of the spin around the interfacial spin-orbit field, leading to a loss of spin angular momentum to the spin-orbit field, referred to as spin memory loss.

## Interfacial and bulk spin torques

Equation (9) describes the charge current as continuous across the interface, ensuring that the flux of particles is conserved. However, the spin current is discontinuous, as the interface scattering causes a portion of the spin angular momentum to be transferred to the effective field. This angular momentum transfer can be described in terms of the nonequilibrium spin density generated at the interface from the incident out-of-plane and in-plane currents, which couples to the effective field through the effective exchange interaction. Since the total angular momentum must be conserved, the effective field at the interface experiences a torque. At $z = 0$, only the transmitted parts of the distributions are present; thus, the ensemble-averaged spin density reads

12 $$\begin{aligned} \langle \varvec{s}\rangle= & \frac{-m_e}{e\hbar A} \sum _{\varvec{k_\Vert }\in \textrm{FS}} \frac{1}{k_z}\operatorname {Tr}\left[ \varvec{\hat{\sigma }}\left( {\mathcal {T}}^{\uparrow \uparrow }_{\varvec{k}}\hat{p}_{\varvec{k}}^\uparrow \left( \hat{g}_{\varvec{k}}^{\textrm{N}} + \hat{g}_{\varvec{k}}^{\textrm{F}}\right) \hat{p}_{\varvec{k}}^\uparrow +{\mathcal {T}}^{\downarrow \downarrow }_{\varvec{k}}\hat{p}_{\varvec{k}}^\downarrow \left( \hat{g}_{\varvec{k}}^{\textrm{N}} + \hat{g}_{\varvec{k}}^{\textrm{F}}\right) \hat{p}_{\varvec{k}}^\downarrow +{\mathcal {T}}^{\uparrow \downarrow }_{\varvec{k}}\hat{p}_{\varvec{k}}^\uparrow \left( \hat{g}_{\varvec{k}}^{\textrm{N}}+ \hat{g}_{\varvec{k}}^{\textrm{F}}\right) \hat{p}_{\varvec{k}}^\downarrow \right. \right. \nonumber \\ & \left. \left. +({\mathcal {T}}^{\uparrow \downarrow }_{\varvec{k}})^*\hat{p}_{\varvec{k}}^\downarrow \left( \hat{g}_{\varvec{k}}^{\textrm{N}}+ \hat{g}_{\varvec{k}}^{\textrm{F}}\right) \hat{p}_{\varvec{k}}^\uparrow \right) \right] . \end{aligned}$$

Assuming the effective field consists of both the magnetization and a spin-orbit field, the interfacial torque can be separated into two contributions describing the torque acting on each. The torque exerted on the magnetization is given by^25,26^

13 $$\begin{aligned} {\varvec{\tau }^\textrm{mag}} = -\frac{2J_{m}}{\hbar }\langle \varvec{s}\rangle \times \varvec{m} = \left( \tilde{\Gamma }^{\textrm{mag}}_{+} - \tilde{\Gamma }^{\textrm{mag}}_{\uparrow \downarrow }\right) \overline{{\varvec{\mu }_{s}}} -\left( {\varvec{\gamma }^{\textrm{mag},F}_{-}}+{\varvec{\gamma }^{\textrm{mag},N}_{-}}\right) E_x -P\left( \tilde{\gamma }^{\textrm{mag},F}_{+} - \tilde{\gamma }^{\textrm{mag},F}_{\uparrow \downarrow } \right) \varvec{m}E_x, \end{aligned}$$

where $\overline{{\varvec{\mu }_{s}}}={\varvec{\mu ^F}_{s}}+{\varvec{\mu ^N}_{s}}$. The first term describes the torque from the transmitted out-of-plane spin currents. The second term accounts for the REE spin density contribution produced by in-plane charge currents at the interface, while the third term describes the SREE contribution from the in-plane spin currents. The torque on the spin-orbit part of the effective field describes a transfer of angular momentum from the spin current to the crystal lattice mediated through the spin-orbit and Coulomb interactions^26^. The crystal lattice acts like an infinite reservoir of angular momentum, thus, this torque can be considered a parasitic loss of spin current.

In addition to the interfacial torques, the magnetization in the bulk of the FM experiences torque due to the transverse spin current. The transverse spin currents in the FM transfer their angular momentum to the magnetization, resulting in STTs. Assuming the transverse spin currents fully decay in the bulk, the total spin torque acting on the magnetization in the bulk is given by the transverse spin current on the FM side of the interface: $\tau ^\textrm{FM} = (I-\varvec{m}\otimes \varvec{m})\varvec{j_{zs}}(0^+)$, where the operator $(I-\varvec{m}\otimes \varvec{m})$ removes the components longitudinal to the magnetization. Suppose the FM layer is thin, and the transverse spin currents do not fully decay. In that case, the bulk torque is obtained from the loss of transverse spin currents across the layer

14 $$\begin{aligned} \varvec{\tau }^{\textrm{FM}}_{\varvec{s}} = -\sigma \int _{0^+}^{d_{F}}dz \left[ \frac{\varvec{\mu _s}(z) \times \varvec{m}}{\lambda _{J}^2}+\frac{\varvec{m}\times (\varvec{\mu _s}(z) \times \varvec{m})}{\lambda _{\phi }^2}\right] , \end{aligned}$$

where $d_F$ is the thickness of the FM layer.

In a NM/FM bilayer the total torque can be split up into five different contributions: ${\varvec{\tau }}^{\textrm{tot}} = {\varvec{\tau }}^{\textrm{MCT}}_{\varvec{{\mu }}} + {\varvec{\tau }}^{\textrm{SOF}}_{\varvec{E}} + {\varvec{\tau }}^{\textrm{SOP}}_{\varvec{E}} + {\varvec{\tau }}^{\textrm{REE}}_{\varvec{E}} + {\varvec{\tau }}^{\textrm{SREE}}_{\varvec{E}}$. The first contribution $\varvec{\tau ^\textrm{MCT}_{\mu }}$ is the torque arising from the spin accumulation, which is the torque from the MCT modified by the interfacial SOC:

15 $$\begin{aligned} {\varvec{\tau }^\textrm{MCT}_{\mu }} = \left( \tilde{\Gamma }^{\textrm{mag}}_{+}-\tilde{\Gamma }^{\textrm{mag}}_{\uparrow \downarrow }\right) \overline{{\varvec{\mu }_{s}}} + (I-\varvec{m}\otimes \varvec{m})\big (\tilde{G}_{+}\Delta {\varvec{\mu }_{s}}-{\varvec{G}_{-}}\Delta \mu _{c} + \tilde{G}_{\uparrow \downarrow }{\varvec{\mu }_{s}^{\textrm{N}}} - \tilde{\Gamma }_{\uparrow \downarrow }{\varvec{\mu }_{s}^{\textrm{F}}}\big ) \end{aligned}$$

The spin accumulation depends on the out-of-plane currents in the bulk. Therefore, Eq. (15) captures the contribution to the torque from the bulk currents which are injected into the FM, such as the SHE current. The remaining four contributions originate from the in-plane charge and spin currents at either side of the interface. The first two are the transverse spin currents at the FM side of the interface generated through SOF and SOP, which are given by

16 $$\begin{aligned} \varvec{\tau }^{\textrm{SOF}}_{\varvec{E}} = (I-\varvec{m}\otimes \varvec{m})(-\Delta {\varvec{\sigma }_{-}}E_x), \quad \text {and} \quad {\varvec{\tau }}^{\textrm{SOP}}_{\varvec{E}} = (I-\varvec{m}\otimes \varvec{m})[-P(\tilde{\sigma }^F_{+}-\tilde{\sigma }^F_{\uparrow \downarrow })\varvec{m}E_x], \end{aligned}$$

respectively. The latter two are the contributions to the spin density at the interface from the REE and SREE, given by

17 $$\begin{aligned} {\varvec{\tau }}^{\textrm{REE}}_{\varvec{E}} = -\left( {\varvec{\gamma }^{\textrm{mag},F}_{-}}+{\varvec{\gamma }^{\textrm{mag},N}_{-}}\right) E_x, \quad \text {and} \quad {\varvec{\tau }}^{\textrm{SREE}_E} =-P\left( \tilde{\gamma }^{\textrm{mag},F}_{+}-\tilde{\gamma }^{\textrm{mag},F}_{\uparrow \downarrow } \right) \varvec{m}E_x, \end{aligned}$$

respectively.

## Current-induced torques in CIP NM/FM bilayers

We consider a NM/FM bilayer with an in-plane electric field $\varvec{E}$. At the interface, an exchange and Rashba spin-orbit field contribute to the effective field. The direction of the effective field at the interface is then given by $\varvec{b}(\varvec{k_\Vert }) = (u_m\varvec{m} + u_R\varvec{\hat{k}}\times \varvec{\hat{z}})/\Vert u_m\varvec{m} + u_R\varvec{\hat{k}}\times \varvec{\hat{z}}\Vert$, where $u_R$ is the dimensionless magnitude of the Rashba SOC. The dimensionless majority/minority potential barrier magnitude is then given by $u^{\uparrow /\downarrow }(\varvec{k_\Vert }) = u_0 \mp \Vert u_m\varvec{m} + u_R\varvec{\hat{k}}\times \varvec{\hat{z}}\Vert$. The parameters $u_m$ and $u_R$ represent the strengths of the interfacial exchange interaction and Rashba spin–orbit coupling, respectively, with their ratio $u_m/u_R$ reflecting the balance between exchange- and SOC-dominated interfacial scattering. Although these quantities cannot be directly obtained from experiments or *ab initio* calculations due to the idealized delta function model, they can be treated as fitting parameters to gain physical insight into the role of these interactions. We solve the continuity equations for the currents (11) with Eq. (10) using Eq. (9) as boundary condition for the currents at either side of the NM/FM interface and compute the torques acting on the magnetization. At external interfaces we assume zero spin and charge currents.

**Fig. 4:**
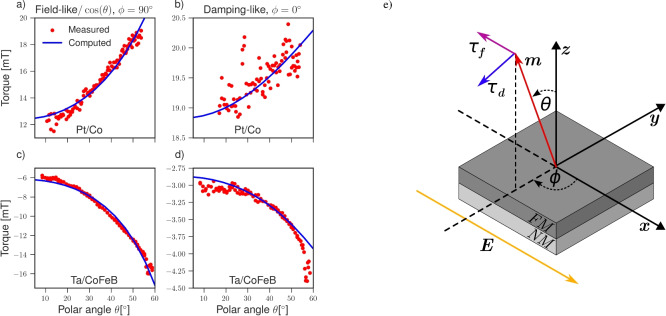
The angular dependence of the SOTs in NM/FM bilayers. The system geometry considered is depicted in (**e**), where the polar ($\theta$) and azimuthal angle ($\phi$) describe the magnetization direction. (**a**,**c**) Show the FL torque for $\phi = 90^\circ$, while (b, d) show the DL torque for $\phi = 0^\circ$. (**a**,**b**) and (**c**,**d**) show computed torques fitted to experimental data for a Pt(3 nm)/Co (0.6 nm) and Ta(3 nm)/CoFeB(0.9 nm) bilayer, respectively. The experimental data are taken from^15^, the bulk and interface parameters used are presented in Table 1a and b, respectively.

Figure 4e depicts the geometry of the system considered, where the magnetization is described by the polar and azimuthal angles $\theta$ and $\phi$, respectively, such that $\varvec{m} = [\cos (\phi )\sin (\theta ), \sin (\phi )\sin (\theta ),\cos (\theta )]$. When $\phi = 0^\circ$, the DL torque simplifies to $\varvec{\tau _d}(\varvec{m}) = \tau _d(\varvec{m})\varvec{\hat{y}}$, and when $\phi = 90^\circ$, the FL torque simplifies to $\varvec{\tau _f}(\varvec{m}) = \tau _f(\varvec{m})\cos (\theta )\varvec{\hat{x}}$. Varying the polar angle in each plane allows to investigate the angular dependence of the DL and FL torque magnitudes $\tau _d(\varvec{m})$ and $\tau _f(\varvec{m})$, respectively. We choose parameters corresponding to Pt and Co for the NM and FM layers, shown in Table 1a. To determine proper parameters for the interface, we fit the computed torques to reported experimental data^15^, for a Pt($3\,\hbox {nm}$ )/Co($0.6\,\hbox {nm}$)/AlO$_x$($1.6\,\hbox {nm}$) system. The resistivity of the entire three layer system was reported to be $36\,\upmu \Omega \hbox {/cm}$, and the width of the sample was $1\,\upmu \hbox {m}$ with a $1.136\,\textrm{mA}$ current, using Ohm’s law the applied electric field is estimated to be $7.86\,\hbox {MV/cm}$. The torques are computed using the estimated electric field with the bulk conductivities of Pt and Co from Table 1a. To directly compare the data we convert the computed torques from units of current density to units of magnetic flux density by multiplying with $-\mu_B/(e\gamma M_sd_{F})$, where $\gamma$ is the gyromagnetic ratio, $\mu _B$ is the Bohr magneton, $M_s$ is the saturation magnetization, and $d_{F}$ is the thickness of the FM layer. For Pt/Co, a Co saturation magnetization of $M_s \approx 600$ kA/m was reported^28^.

**Table 1:** Parameters for the bulk (a) and for the interface (b). Bulk material parameters were taken from reported values in the literature or extracted from experimental or *ab initio* data. The momentum relaxation times were computed from the conductivities and Fermi wave numbers using the Drude model.

a						b			
Parameter	Pt	Ta	Co	CoFeB	Units	Parameter	Pt/Co	Ta/CoFeB	Units
$\sigma$	7^29^	0.5^30^	5^31^	3.3^32^	$\mathrm {MSm^{-1}}$	$u_0$	0.06	0.07	1
$\alpha _\textrm{SH}$	0.19^29^	$-0.15$^33^			1	$u_m$	0.28	1.56	1
P			0.36^31^	0.56^32^	1	$u_R$	0.22	$-1.18$	1
$\lambda _\textrm{sf}$	1.4^29^	1.9^30^	38^31^	12^31,32^	nm	$k_F$	16	15	nm$^{-1}$
$\lambda _{J}$			1.2^34^	1.2^34^	nm	$\tau ^\textrm{F}$	1.28	1.04	fs
$\lambda _{\phi }$			1.8^34,35^	2.0	nm	$\tau ^\textrm{N}$	1.80	1.56	fs

Figure 4a,b show the best fit achieved by setting $k_F = 16 \,\hbox {nm}^{-1}$, and using $u_0$, $u_m$ and $u_R$ as fitting parameters. The fitted values were obtained using the nonlinear least squares method, and are presented in Table 1b. The FL and DL torque magnitude fit agrees exceptionally well with the experimental data, demonstrating that the model accounts for the strong FL torque and the higher-order torques reported in the literature. Depending on the angle, the FL torque magnitude exhibits a strong angular dependence, while the angular dependence of the DL torque magnitude is less pronounced.

Experimental data for a Ta ($3.0\,\hbox {nm}$ )/CoFeB($0.9\,\hbox {nm}$) bilayer with an MgO($2.0\,\hbox {nm}$) capping layer were also reported^15^. With a resistivity of $184\,\upmu \Omega \hbox {cm}$, a current of $500\,\upmu \hbox {A}$, and sample width of $1\,\upmu \hbox {m}$, the applied in-plane electric field is approximately $15.7 \,\hbox {MV/cm}$. Figure 4c,d show the best fit achieved with the bulk and interface parameters presented in Table 1a and 1b, respectively, with a reported CoFeB saturation magnetization of $M_s \approx 810$ kA/m^36^. The fit agrees well with the reported experimental data, except for the DL torque for small and large polar angles. This system’s FL torque dominates and has a strong angular dependence. In contrast with the previous system, the signs of the torques are reversed, which is consistent with the negative sign of the Ta spin Hall angle and Rashba SOC.

For both the Pt/Co and Ta/CoFeB systems, we have ignored the interfaces introduced by the insulating capping layer and substrate used in the experimental setup (SiO$_2$). The choice of substrate for the bilayer has been shown to significantly affect the current-induced torques, when the NM layer is thin^37^, suggesting that this interface can also be an additional spin current source. Furthermore, the interface between the FM and capping oxide layers can also generate spin currents^38^. Both of these interfaces could contribute to the experimental results from^15^, which could explain the discrepancies in the fit for the Ta/CoFeB system. Furthermore, the interface model assumes perfectly sharp and flat interfaces, thereby neglecting the effects of interfacial roughness and intermixing. Roughness can increase incoherent spin scattering and introduce spatial variations in the Rashba spin–orbit field, which can in turn reduce interface transparency and diminish the overall spin–torque efficiency. However, scattering due to interface roughness could potentially generate weak spin currents similarly to the SHE due to impurity scattering in the bulk^39^. Intermixing, by contrast, gives rise to a finite-thickness interfacial layer whose transport properties differ from those of the adjacent bulk layers. Such a mixed layer can enhance the interfacial SOC, thereby increasing both the SOTs and the spin-memory loss^40,41^. A comparatively thick intermixing interface region has been reported for the Ta/CoFeB interface^42^, which is in clear conflict with the assumptions of the idealized interface model. Strain at the interfaces could also introduce unaccounted-for modifications to the SOC and SOTs; for instance, the bulk Dresselhaus SOC in materials with zinc-blende structure changes its form when strain is introduced at the interface^43^.

Although interface disorder and strain can potentially strongly influence the SOTs, their impact on the angular dependence remains unclear, as experimental studies on this aspect are relatively scarce. However, annealing can modify both the magnitude and angular dependence of the torques. In Ta/CoFeB bilayers, a pronounced increase in the angular dependence of the damping-like torque was observed once the annealing temperature exceeded $270^\circ \textrm{C}$^44^. Since higher annealing temperatures have been demonstrated to broaden the intermixing region at the Ta/CoFeB interface^45^, this strongly suggests that a thick intermixing interface region may explain the discrepancy between the fitted and measured angular dependence of the damping-like torque in the Ta/CoFeB system, as the experimental data were obtained using an annealing temperature of $300^\circ \textrm{C}$. Modeling the interface as a finite-thickness potential barrier in the Hamiltonian could, in principle, capture the effects of such intermixing, but this lies beyond the scope of the present work and is left for future studies. Nonetheless, the good agreement of the fits with the experimental data provides a strong evidence for the presence of an interfacial Rashba SOC at the Pt/Co and Ta/CoFeB interfaces.

## Modification of SOTs by interfacial Rashba interaction

**Fig. 5:**
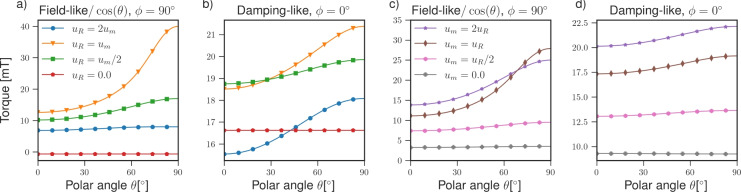
The angular dependence of the spin torque for different strengths of the: Rashba SOC $u_R$ (**a**,**b**), magnetic exchange interaction $u_m$ (**c**,**d**). The calculations were performed for the Pt/Co system from the previous section using the parameters presented in Table 1.

To investigate the role of the Rashba SOC and magnetic exchange interaction at the interface, we compute the torques for various values of $u_R$ and $u_m$ using the parameters for Co/Pt; the resulting torque magnitudes are shown in Fig. 5.

The FL and DL torques strongly depend on the interaction strengths. The angular dependence and the magnitude of the field/like torque are most pronounced, when the interfacial SOC and magnetic exchange interactions are equal. The angular dependence is less pronounced for higher or lower interaction strengths, and the magnitude is reduced. The DL torque shows a similar dependence, except that the DL torque is most pronounced for $u_m > u_R$. The angular dependence of the FL and the DL torques disappears, when either $u_R = 0$ or $u_m = 0$.

Similar results were obtained using two-dimensional free electron and tight binding models with Rashba and magnetic exchange interactions^19^. The angular dependence was attributed to the deformation of the Fermi surface spin texture, when the Rashba and magnetic exchange interactions were comparable in strength. An intrinsic contribution from the Fermi sea to the DL torque was also identified. The three-dimensional scattering model presented in this work differs significantly from two-dimensional models as it considers just the electrons incident on the interface interacting with the effective field and the resulting outbound currents. In contrast, two-dimensional models treat the interface as the entire system. Consequently, only the electrons inside the interface are considered, and there is no scattering at the interface. In the scattering model, the contribution from the deformation of the Fermi sea is captured through the strong momentum dependence of the majority/minority potential barriers $u^{\uparrow /\downarrow }_{\varvec{k}}$ and spin-projection operators $\hat{p}^{\uparrow /\downarrow }_{\varvec{k}}$, when $u_R$ and $u_m$ are comparable in magnitude. However, the Fermi sea contribution is not captured as it originates in the modification of the wavefunction by the electric field, which is not considered here. Nonetheless, the scattering approach accounts for the additional effects arising from the in-plane and out-of-plane spin and charge currents on either side of the interface interacting with the SOC.

**Fig. 6:**
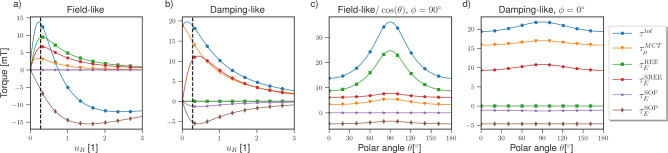
The different contributions to the total torque as a function of: the Rashba SOC $u_R$ (**a**,**b**), the polar angle of the magnetization direction $\theta$ (**c**,**d**). For (**a**,**b**), the magnetization points out of the plane, and the dashed line marks where $u_R = u_m$. The calculations were performed for the Pt/Co system from the previous section using the parameters presented in Table 1.

Figure 6a,b show the different contributions to the total spin torque captured by the three-dimensional scattering model as a function of the Rashba interaction strength $u_R$. As expected, within the limit of vanishing SOC, the additional contributions arising from the in-plane currents vanish, and only the MCT contribution from the SHE through the accumulations remains. However, in contrast to typical two-dimensional models of the REE, the contributions from the in-plane currents do not have a linear dependence on the Rashba interaction strength. Instead, we observe that most of these contributions reach a maximum when $u_R \approx u_m$, with the exception of the SOP, followed by a decaying magnitude with increasing $u_R$. The loss of spin angular momentum to the lattice appears in the SHE contribution $\tau _\mu ^{\textrm{MCT}}$ to the field-like torque, which decreases with increasing $u_R$ due to enhanced spin–memory loss of the bulk SHE spin current at the interface. In contrast, the SOC-driven contributions initially grow with increasing $u_R$ as the in-plane currents are more efficiently converted to out-of-plane current. These contributions peak near $u_R \approx u_m$, where interfacial precession around the magnetization most effectively generates transverse spin components. At larger $u_R$, transmission rapidly decreases while reflection increases, causing the SOP contribution, which relies only on spin-dependent reflections, to become the dominant contribution to the field-like torque for $u_R > u_m$, reaching a maximum near $u_R \approx 1.5u_m$. The SOF only contributes to the DL torque, while the SOP contributes to both. Both the SOF and SOP torques oppose the other contributions. Consistent with two-dimensional models, the REE contributes only to the FL torque; however, the SREE contributes to both and constitutes the most significant contribution to the DL torque apart from the MCT torque. Figure 6c,d show the angular dependence of the different torque terms, which exhibit the same angular dependence to varying degrees. We observe that the REE is predominantly responsible for the strong angular dependence of the FL torque magnitude. The MCT torque also obtains an angular dependence as it becomes modified by the SOC.

## Interface generated spin currents in CIP FM/NM/FM trilayers

**Fig. 7:**
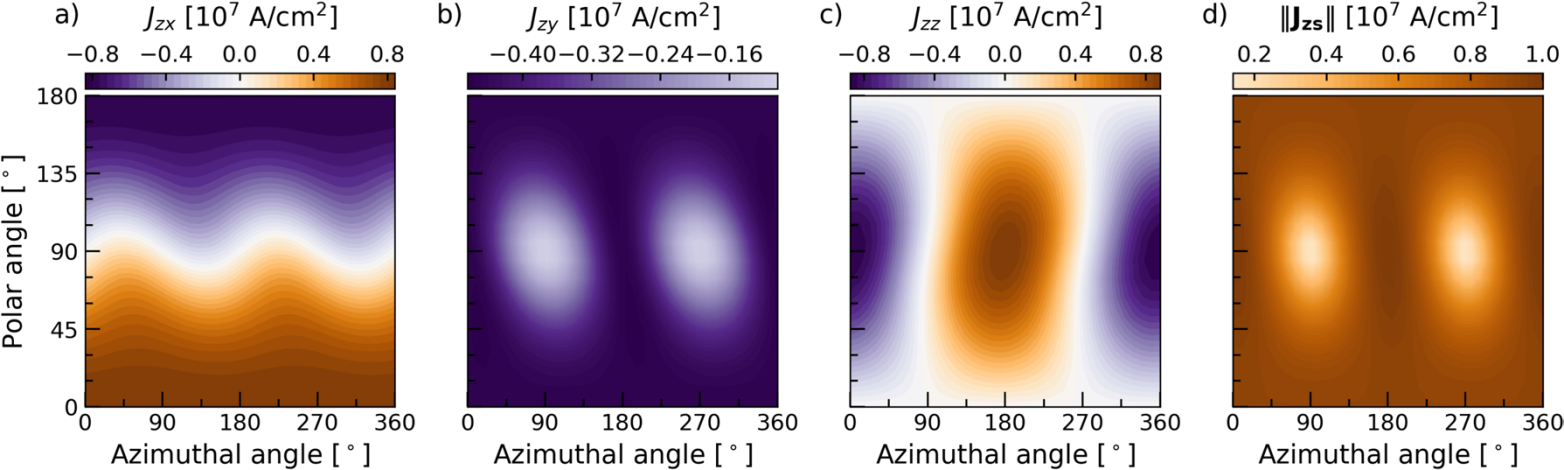
Out-of-plane spin current generated at the Pt side of a Co/Pt interface as a function of the magnetization direction. (**a**–**c**) Show the x, y, and z polarization components, respectively, while (**d**) shows the total spin current magnitude. The calculations were performed using the Co/Pt parameters presented in Table 1.

We consider FM/NM/FM trilayers with an in-plane electric field and investigate the spin currents generated by the additional FM/NM interface. At the NM/FM bottom interface, the SOF and SOP generate out-of-plane spin currents which propagate through the NM layer. Figure 7 shows the angular dependence of the spin currents generated at the Pt side of a Co/Pt interface with a $10^6 \,\hbox {Vm}^{-1}$ in-plane electric field. In general, the interface-generated spin currents exhibit a strong dependence on the magnetization, and depending on the magnetization direction, all spin polarization components can be generated with comparable magnitude. As different polarization components dominate in various directions the polarization of the currents can be controlled with the magnetization direction. A magnetization along $\pm z$ generates spin currents with a strong polarization component along ${\mp } x$. Conversely, a magnetization along the current direction $\pm x$ yields a strong component along ${\mp } z$. Moreover, a magnetization along $\pm y$ yields a predominantly $-y$ polarized spin current.

**Fig. 8:**
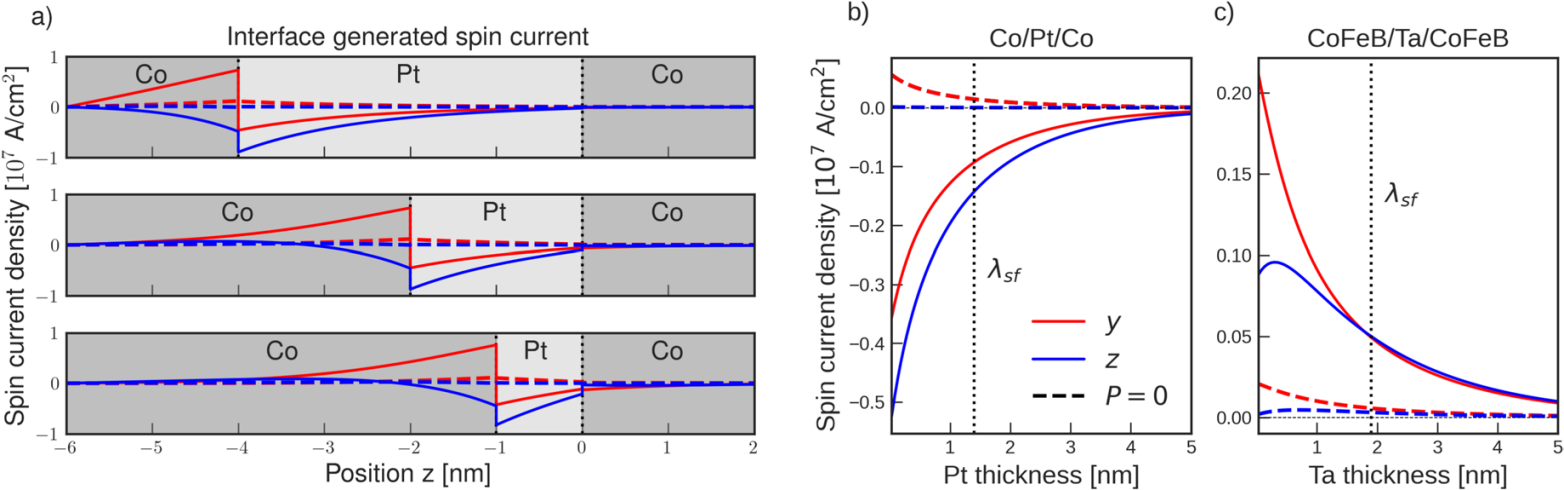
(**a**) The distribution of the spin current density generated at the lower Co/Pt interface in a CIP Co/Pt/Co trilayer. (**b**) The spin current density at the Pt/Co interface ($z=0$) for a Co/Pt/Co trilayer. (**c**) The same as (**b**) for a CoFeB/Ta/CoFeB trilayer. Both FM layer magnetizations are along $\varvec{\hat{x}}$. When $P=0$, only the SOF current is generated at the bottom interface. The calculations were performed using the Co/Pt and Ta/CoFeB parameters presented in Table 1.

Figure 8a shows the spin currents generated at the bottom Co/Pt interface for different thicknesses of the Pt layer. The spin currents are generated at the lower interface and decay across the bulk of the NM layer due to bulk spin-flip processes. By reducing the Pt thickness, more of the spin current reaches the upper Pt/Co layer interface. For $P=0$, the interface-generated spin currents are drastically diminished, demonstrating that SOP is mainly responsible for the interface currents generated at the Co/Pt interface. The remaining contribution arises from the SOF spin current, which is independent of the in-plane spin currents and instead scales with the difference in momentum relaxation times across the interface. Consequently, a strong conductivity mismatch can significantly enhance the SOF contribution, providing a plausible explanation for the pronounced interface-generated spin currents reported in Pt/W^46^. Figure 8b and c show the spin current at the upper interface for a Co/Pt/Co and CoFeB/Ta/CoFeB trilayer as a function of the Pt and Ta thicknesses, respectively. For both systems, the *z* and *y* polarized currents are comparable in magnitude and increase with decreasing spacer thickness. In the Co/Pt/Co system, the *z*-component is the most pronounced one of all components. For the CoFeB/Ta/CoFeB system at thicknesses above $\lambda _{sf}$, the *y* and *z*-components are comparable in magnitude, while at thicknesses below $\lambda _{sf}$, the *y*-component is the most pronounced one. Maximizing the contribution to the torque from these currents necessitates a NM spacer thickness below $\lambda _{sf}$, opposite to what is required to maximize the contribution from the SHE.

For miniaturization of SOT-MRAM, the switching of *x* and *z* states is desirable as they do not require widening of the stack to pin the magnetization reliably. The shape anisotropy of the SOT track can pin a magnetization state along *x*, while a *z*-state can take advantage of the perpendicular interface anisotropy. The interface-generated currents can potentially enable field-free switching for sufficiently thin NM spacer thicknesses. A *x*-type MTJ would then require the bottom FM layer magnetization to be oriented along $\pm z$, while a *z*-type would require a magnetization along $\pm x$. Reliable field-free switching of a perpendicular magnetization state in an FM/NM/FM trilayer was demonstrated by Baek et al.^47^, where the magnetization of the bottom FM layer was along the current direction, and the perpendicular switching was attributed to SOF and SOP. Later, Ryu et al. demonstrated that the efficiency can be improved by using a tilted bottom layer magnetization, thus exploiting all three components of the polarization^48^. Recently, it was also demonstrated using micromagnetic simulations that CIP-trilayer-generated out-of-plane polarized spin currents can be utilized to induce large-amplitude GHz oscillations in SHNOs without requiring a bias field^49^. The oscillations were shown to be self-sustained with a ratio of *z*- to *y*-polarized currents as low as $4\%$. The applications are promising; however, experimental confirmations of such oscillations are still lacking.

In addition to FM/NM/FM trilayers, several bilayer systems using materials beyond conventional heavy metals for spin current generation have been reported to exhibit large unconventional torques with both bulk and interfacial origins. For example, Weyl semimetals such as TaIrTe$_4$^50^, and collinear and non-collinear antiferromagnets such as epitaxial FeSn and Mn$_3$Sn^51,52^, respectively, can generate out-of-plane polarized spin currents in their bulk, which induce unconventional torques in adjacent ferromagnetic layers. Similarly, the interfaces between Py and the insulator EuS^53^, or the non-collinear antiferromagnet $\gamma$-IrMn$_3$^54^, have also been shown to produce large out-of-plane spin-polarized currents and unconventional torques. The spin drift-diffusion model can be extended to capture such bulk contributions by modifying the spin Hall conductivity tensor to include *z*-polarized spin currents; the resulting torques have been shown to enable field-free switching^55^. Accounting for the reported interfacial contributions would require an *x*-polarized in-plane spin current in the NM layer, which could generate unconventional torques through SOP and the SREE, or involve a form of interfacial spin–orbit coupling other than the Rashba SOC considered in this work.

## Conclusions

A description of the spin and charge currents at NM/FM interfaces with strong interfacial exchange and SOC relative to bulk splitting has been presented. The model is derived from considering a three-dimensional delta function potential barrier and treats interfacial SOC nonperturbatively, enabling the exploration of strong SOC regimes. By using expressions for the currents as boundary conditions for the drift-diffusion equations, the interactions between bulk currents and the interface, and between multiple NM/FM layers and interfaces are treated. Additionally, the SOTs acting on the magnetization of the FM layers can be evaluated, capturing contributions arising from the spin accumulation generated by in-plane currents through the SOC. Using the Rashba form of interfacial SOC, we demonstrate that the experimentally measured angular dependence of SOTs in Co/Pt and Ta/CoFeB bilayers can be reproduced by fitting the interface parameters introduced by the model. The strong angular dependence is most pronounced, when the interfacial exchange and spin-orbit interaction strengths are comparable. Moreover, the REE is mainly responsible for the substantial magnitude and angular dependence of the FL torque, which is consistent with two-dimensional Rashba models. Unlike two-dimensional models, the three-dimensional description accounts for out-of-plane currents generated by in-plane currents through scattering from the interfacial spin-orbit field. The interface-generated currents can be transported through the NM layer, reaching other adjacent FM layers. In FM/NM/FM trilayers with a pinned bottom layer magnetization, if the NM thickness is thinner or comparable to the spin-flip length, the interface-generated currents can switch the upper layer deterministically. For an in-plane current along the x-axis, spin currents with polarization along x and z are generated, when the bottom layer magnetization is oriented along the z- or x-axis, respectively.

## Data Availability

The datasets generated during and/or analyzed during the current study are available from the corresponding author on reasonable request.

## Appendix: Interface conductance and conductivity tensors

The conductance tensors are given by the following Fermi surface integrals:

18a $$\begin{aligned}&G^l_{+} = \int \frac{d\varvec{k_\Vert }}{(2\pi )^2}\left( \frac{k_x}{k_F}\right) ^l \left[ {\mathcal {G}}^{\uparrow \uparrow }(\varvec{k_\Vert }) + {\mathcal {G}}^{\downarrow \downarrow }(\varvec{k_\Vert })\right] \end{aligned}$$

18b $$\begin{aligned}&{\varvec{G}^l_{-}} = \int \frac{d\varvec{k_\Vert }}{(2\pi )^2}\left( \frac{k_x}{k_F}\right) ^l \left[ {\mathcal {G}}^{\uparrow \uparrow }(\varvec{k_\Vert }) - {\mathcal {G}}^{\downarrow \downarrow }(\varvec{k_\Vert })\right] \varvec{b}(\varvec{k_\Vert }) \end{aligned}$$

18c $$\begin{aligned}&\tilde{G}^l_{+} = \int \frac{d\varvec{k_\Vert }}{(2\pi )^2}\left( \frac{k_x}{k_F}\right) ^l \left[ {\mathcal {G}}^{\uparrow \uparrow }(\varvec{k_\Vert }) + {\mathcal {G}}^{\downarrow \downarrow }(\varvec{k_\Vert })\right] \varvec{b}(\varvec{k_\Vert })\otimes \varvec{b}(\varvec{k_\Vert }), \end{aligned}$$

18d $$\begin{aligned}&\tilde{G}^l_{\uparrow \downarrow } = \int \frac{d\varvec{k_\Vert }}{(2\pi )^2}\left( \frac{k_x}{k_F}\right) ^l[\varvec{b}(\varvec{k_\Vert })]_\times \left( 2\operatorname {Re}[{\mathcal {G}}^{\uparrow \downarrow }(\varvec{k_\Vert })][\varvec{b}(\varvec{k_\Vert })]_\times - 2\operatorname {Im}[{\mathcal {G}}^{\uparrow \downarrow }(\varvec{k_\Vert })] \right) \end{aligned}$$

18e $$\begin{aligned}&\tilde{\Gamma }^l_{\uparrow \downarrow } = \int \frac{d\varvec{k_\Vert }}{(2\pi )^2}\left( \frac{k_x}{k_F}\right) ^l[\varvec{b}(\varvec{k_\Vert })]_\times \left( 2\operatorname {Re}[{\mathcal {T}}^{\uparrow \downarrow }(\varvec{k_\Vert })][\varvec{b}(\varvec{k_\Vert })]_\times - 2\operatorname {Im}[{\mathcal {T}}^{\uparrow \downarrow }(\varvec{k_\Vert })]\right) \end{aligned}$$

The vector operations are defined as $(\varvec{v}\otimes \varvec{v})_{ij} = v_iv_j$, and $([\varvec{v}]_\times )_{ij} = \varepsilon _{ikj}v_k$, for a vector $\varvec{v}$, where $\varepsilon _{ijk}$ is the Levi-Civita symbol. The superscript *l* denotes if the conductance contains a $k_x/k_F$ factor due to the shift of the Fermi surface ($l = 1$) or not ($l=0$). We define the interface conductivity tensors as follows:

19 $$\begin{aligned} \sigma ^{F/N}_{+} = G^1_{+}v_F\tau ^{F/N}, \quad {\varvec{\sigma }^{F/N}_{-}} = \varvec{G^1_{-}}v_F\tau ^{F/N}, \quad \tilde{\sigma }^{F/N}_{+} = \tilde{G}^1_{+}v_F\tau ^{F/N}, \quad \tilde{\sigma }^{F/N}_{\uparrow \downarrow } =\tilde{G}^1_{\uparrow \downarrow }v_F\tau ^{F/N}, \quad \tilde{\gamma }^{F/N}_{\uparrow \downarrow } = \tilde{\Gamma }^1_{\uparrow \downarrow }v_F\tau ^{F/N} \end{aligned}$$

The interface torque conductances (torkances) and conductivities (torkivities) read:

20a $$\begin{aligned}&{\varvec{\Gamma }^{\textrm{mag},l}_{-}} = 2u_m\int \frac{d\varvec{k_\Vert }}{(2\pi )^2}\left( \frac{k_x}{k_F}\right) ^l\frac{k_F}{k_z}\left[ {\mathcal {T}}^{\uparrow \uparrow }(\varvec{k_\Vert }) - {\mathcal {T}}^{\downarrow \downarrow }(\varvec{k_\Vert })\right] \varvec{m}\times \varvec{b}(\varvec{k_\Vert }) \end{aligned}$$

20b $$\begin{aligned}&\tilde{\Gamma }^{\textrm{mag},l}_{+} = 2u_m\int \frac{d\varvec{k_\Vert }}{(2\pi )^2}\left( \frac{k_x}{k_F}\right) ^l\frac{k_F}{k_z} \left[ {\mathcal {T}}^{\uparrow \uparrow }(\varvec{k_\Vert }) + {\mathcal {T}}^{\downarrow \downarrow }(\varvec{k_\Vert })\right] [\varvec{m}\times \varvec{b}(\varvec{k_\Vert })]\otimes \varvec{b}(\varvec{k_\Vert }) \end{aligned}$$

20c $$\begin{aligned}&\tilde{\Gamma }^{\textrm{mag},l}_{\uparrow \downarrow } = 2u_m\int \frac{d\varvec{k_\Vert }}{(2\pi )^2}\left( \frac{k_x}{k_F}\right) ^l\frac{k_F}{k_z} [\varvec{m}]_\times \left( 2\operatorname {Re}[{\mathcal {T}}^{\uparrow \downarrow }(\varvec{k_\Vert })][\varvec{b}(\varvec{k_\Vert })]_\times [\varvec{b}(\varvec{k_\Vert })]_\times - 2\operatorname {Im}[{\mathcal {T}}^{\uparrow \downarrow }(\varvec{k_\Vert })[\varvec{b}(\varvec{k_\Vert })]_\times ]\right) \end{aligned}$$

20d $$\begin{aligned} {\varvec{\gamma }^{mag,F/N}_{-}} = {\varvec{\Gamma }^{\textrm{mag},1}_{-}}v_F\tau ^{F/N}, \quad \tilde{\gamma }^{mag,F/N}_{+} = \tilde{\Gamma }^{\textrm{mag},1}_{+}v_F\tau ^{F/N}, \quad \tilde{\gamma }^{mag,F/N}_{\uparrow \downarrow } = \tilde{\Gamma }^{\textrm{mag},1}_{\uparrow \downarrow }v_F\tau ^{F/N} \end{aligned}$$

## References

[1] Bychkov, Y. A. & Rashba, É. I. Properties of a 2d electron gas with lifted spectral degeneracy. *JETP Lett.* **39**, 78–81 (1984).

[2] Edelstein, V. Spin polarization of conduction electrons induced by electric current in two-dimensional asymmetric electron systems. *Solid State Commun.* **73**, 233–235 (1990).

[3] D’Yakonov, M. I. & Perel’, V. I. Possibility of orienting electron spins with current. *Sov. J. Exp. Theor. Phys. Lett.* **13**, 467 (1971).

[4] Manchon, A. et al. Current-induced spin-orbit torques in ferromagnetic and antiferromagnetic systems. *Rev. Mod. Phys.* **91**, 035004 (2018).

[5] Han, X., Wang, X., Wan, C., Yu, G. & Lv, X. Spin-orbit torques: Materials, physics, and devices. *Appl. Phys. Lett.* **118**, 120502 (2021).

[6] Song, C. et al. Spin-orbit torques: Materials, mechanisms, performances, and potential applications. *Prog. Mater. Sci.* **118**, 100761 (2021).

[7] Ramaswamy, R., Lee, J. M., Cai, K. & Yang, H. Recent advances in spin-orbit torques: Moving towards device applications. *App. Phys. Rev.* **5**, 031107 (2018).

[8] Shao, Q. et al. Roadmap of spin-orbit torques. *IEEE Trans. Magn.* **57**, 1–39 (2021). 10.48550/arXiv.2104.11459 PMC10091395 37057056

[9] Nguyen, V., Rao, S., Wostyn, K. & Couet, S. Recent progress in spin-orbit torque magnetic random-access memory. *npj Spintronics* **2**, 48 (2024).

[10] Kang, M.-G., Lee, S. & Park, B.-G. Field-free spin-orbit torques switching and its applications. *npj Spintronics* **3**, 8 (2025).

[11] Jiang, S. et al. Spin-torque nano-oscillators and their applications. *App. Phys. Rev.* **11**, 041309 (2024).

[12] Kumar, A. et al. *Mutual Synchronization in Spin-Torque and Spin Hall Nano-oscillators* 143–182 (Springer Nature Switzerland, 2024).

[13] Ning, S., Liu, H., Wu, J. & Luo, F. Challenges and opportunities for spintronics based on spin orbit torque. *Fundam. Res.* **2**, 535–538 (2022). 38933998 10.1016/j.fmre.2022.05.013 PMC11197755

[14] Krizakova, V., Perumkunnil, M., Couet, S., Gambardella, P. & Garello, K. Spin-orbit torque switching of magnetic tunnel junctions for memory applications. *J. Magn. Magn. Mater.* **562**, 169692 (2022).

[15] Garello, K. et al. Symmetry and magnitude of spin–orbit torques in ferromagnetic heterostructures. *Nat. Nanotechnol.* **8**, 587–593 (2013). 23892985 10.1038/nnano.2013.145

[16] Park, E.-S. et al. Strong higher-order angular dependence of spin-orbit torque in W/CoFeB bilayer. *Phys. Rev. B* **107**, 064411 (2023).

[17] Belashchenko, K. D., Kovalev, A. A. & Schilfgaarde, M. First-principles calculation of spin-orbit torque in a Co/Pt bilayer. *Phys. Rev. Mater.* **3**, 011401 (2019).

[18] Ortiz Pauyac, C., Wang, X., Chshiev, M. & Manchon, A. Angular dependence and symmetry of Rashba spin torque in ferromagnetic heterostructures. *App. Phys. Lett.* **102**, 252403 (2013).

[19] Lee, K.-S. et al. Angular dependence of spin-orbit spin-transfer torques. *Phys. Rev. B* **91**, 144401 (2015).

[20] Kim, K.-W., Lee, K.-J., Sinova, J., Lee, H.-W. & Stiles, M. D. Spin-orbit torques from interfacial spin-orbit coupling for various interfaces. *Phys. Rev. B* **96**, 104438 (2017). 29333523 10.1103/PhysRevB.96.104438 PMC5761703

[21] Haney, P. M., Lee, H.-W., Lee, K.-J., Manchon, A. & Stiles, M. D. Current induced torques and interfacial spin-orbit coupling: Semiclassical modeling. *Phys. Rev. B* **87**, 174411 (2013).

[22] Brataas, A., Nazarov, Y. V. & Bauer, G. E. W. Finite-element theory of transport in ferromagnet–normal metal systems. *Phys. Rev. Lett.* **84**, 2481–2484 (2000). 11018915 10.1103/PhysRevLett.84.2481

[23] Brataas, A., Bauer, G. E. W. & Kelly, P. J. Non-collinear magnetoelectronics. *Phys. Rep.* **427**, 157–255 (2006).

[24] Amin, V. P., Haney, P. M. & Stiles, M. D. Interfacial spin–orbit torques. *J. Appl. Phys.* **128**, 151101 (2020). 10.1063/5.0024019 PMC8194107 34121763

[25] Amin, V. P. & Stiles, M. D. Spin transport at interfaces with spin-orbit coupling: Formalism. *Phys. Rev. B* **94**, 104419 (2016). 10.1103/physrevb.94.104420 PMC11561893 39545023

[26] Amin, V. P. & Stiles, M. D. Spin transport at interfaces with spin-orbit coupling: Phenomenology. *Phys. Rev. B* **94**, 104420 (2016). 10.1103/physrevb.94.104420 PMC11561893 39545023

[27] Valet, T. & Fert, A. Theory of the perpendicular magnetoresistance in magnetic multilayers. *Phys. Rev. B* **48**, 7099–7113 (1993). 10.1103/physrevb.48.7099 10006879

[28] Woo, S. et al. Observation of room-temperature magnetic skyrmions and their current-driven dynamics in ultrathin metallic ferromagnets. *Nat. Mater.* **15**, 501–506 (2016). 26928640 10.1038/nmat4593

[29] Zhang, W., Han, W., Jiang, X., Yang, S.-H. & Parkin, S. P. S. Role of transparency of platinum-ferromagnet interfaces in determining the intrinsic magnitude of the spin Hall effect. *Nat. Phys.* **11**, 496–502 (2015).

[30] Montoya, E. et al. Spin transport in tantalum studied using magnetic single and double layers. *Phys. Rev. B* **94**, 054416 (2016).

[31] Piraux, L., Dubois, S., Fert, A. & Belliard, L. The temperature dependence of the perpendicular giant magnetoresistance in Co/Cu multilayered nanowires. *The Eur. Phys. J. B - Condens. Matter Complex Syst.* **4**, 413–420 (1998).

[32] Oshima, H. et al. Perpendicular giant magnetoresistance of CoFeB/Cu single and dual spin-valve films. *J. App. Phys.* **91**, 8105–8107 (2002).

[33] Liu, L. et al. Spin-torque switching with the giant spin Hall effect of tantalum. *Science* **336**, 555–558 (2012). 22556245 10.1126/science.1218197

[34] Ghosh, A., Auffret, S., Ebels, U. & Bailey, W. E. Penetration depth of transverse spin current in ultrathin ferromagnets. *Phys. Rev. Lett.* **109**, 127202 (2012). 23005979 10.1103/PhysRevLett.109.127202

[35] Petitjean, C., Luc, D. & Waintal, X. Unified drift-diffusion theory for transverse spin currents in spin valves, domain walls, and other textured magnets. *Phys. Rev. Lett.* **109**, 117204 (2012). 23005670 10.1103/PhysRevLett.109.117204

[36] Litzius, K. et al. The role of temperature and drive current in skyrmion dynamics. *Nat. Electron.* **3**, 30–36 (2020).

[37] Choi, G. et al. Thickness dependence of spin–orbit torques in Pt/Co structures on epitaxial substrates. *APL Mater.* **10**, 011105 (2022).

[38] Gabor, M. S., Belmeguenai, M. & Miron, I. M. Bulk and interface spin-orbit torques in Pt/Co/MgO thin film structures. *Phys. Rev. B* **109**, 104407 (2024).

[39] Zhou, L., Grigoryan, V. L., Maekawa, S., Wang, X. & Xiao, J. Spin Hall effect by surface roughness. *Phys. Rev. B* **91**, 045407 (2015).

[40] Flores, G. G. B. & Belashchenko, K. D. Effect of interfacial intermixing on spin-orbit torque in Co/Pt bilayers. *Phys. Rev. B* **105**, 054405 (2022).

[41] Gupta, K., Wesselink, R. J. H., Liu, R., Yuan, Z. & Kelly, P. J. Disorder dependence of interface spin memory loss. *Phys. Rev. Lett.* **124**, 087702 (2020). 32167325 10.1103/PhysRevLett.124.087702

[42] Cecot, M. et al. Influence of intermixing at the Ta/CoFeB interface on spin hall angle in Ta/CoFeB/MgO heterostructures. *Sci. Rep.* **7**, 968 (2017). 28428546 10.1038/s41598-017-00994-z PMC5430535

[43] La Rocca, G. C., Kim, N. & Rodriguez, S. Effect of uniaxial stress on the electron spin resonance in zinc-blende semiconductors. *Phys. Rev. B* **38**, 7595–7601 (1988). 10.1103/physrevb.38.7595 9945486

[44] Avci, C. O. et al. Fieldlike and antidamping spin-orbit torques in as-grown and annealed Ta/CoFeB/MgO layers. *Phys. Rev. B* **89**, 214419 (2014).

[45] Lamperti, A., Ahn, S.-M., Ocker, B., Mantovan, R. & Ravelosona, D. Interface width evaluation in thin layered CoFeB/MgO multilayers including Ru or Ta buffer layer by X-ray reflectivity. *Thin Solid Films* **533**, 79–82 (2013).

[46] Karube, S., Tezuka, N., Kohda, M. & Nitta, J. Anomalous spin-orbit field via the Rashba-Edelstein effect at the interface. *Phys. Rev. Appl.* **13**, 024009 (2020).

[47] Baek, S.-H. et al. Spin currents and spin-orbit torques in ferromagnetic trilayers. *Nat. Mater.* **17**, 509–513 (2018). 29555998 10.1038/s41563-018-0041-5

[48] Ryu, J. et al. Efficient spin-orbit torque in magnetic trilayers using all three polarizations of a spin current. *Nat. Electron.* **5**, 217–223 (2022).

[49] Kubler, D. et al. Large-amplitude easy-plane spin-orbit torque oscillators driven by out-of-plane spin current: A micromagnetic study. *Phys. Rev. B* **111**, 054425 (2025).

[50] Liu, Y. et al. Field-free switching of perpendicular magnetization at room temperature using out-of-plane spins from TaIrTe4. *Nat. Electron.* **6**, 732–738 (2023).

[51] Gupta, P. et al. Symmetry enhanced unconventional spin current anisotropy in a collinear antiferromagnet. *Adv. Funct. Mater.* e06816 (2025).

[52] Hu, S. et al. Efficient perpendicular magnetization switching by a magnetic spin Hall effect in a noncollinear antiferromagnet. *Nat. Commun.* **13**, 4447 (2022). 35915121 10.1038/s41467-022-32179-2 PMC9343665

[53] Gupta, P. et al. Generation of out-of-plane polarized spin current in (permalloy, Cu)/EuS interfaces. *Phys. Rev. B* **109**, L060405 (2024).

[54] Kumar, A. et al. Interfacial origin of unconventional spin-orbit torque in Py/-IrMn3. *Adv. Quantum Technol.* **6**, 2300092 (2023).

[55] Pruckner, B., Jørstad, N. P., Goes, W., Selberherr, S. & Sverdlov, V. Field-free magnetization switching in SOT-MRAM devices with noncollinear antiferromagnets. *Microelectron. Eng.* **300**, 112372 (2025).

